# Inhibition of *Listeria monocytogenes* in Fresh Cheese Using Chitosan-Grafted Lactic Acid Packaging

**DOI:** 10.3390/molecules21040469

**Published:** 2016-04-08

**Authors:** Laura N. Sandoval, Monserrat López, Elizabeth Montes-Díaz, Andres Espadín, Alberto Tecante, Miquel Gimeno, Keiko Shirai

**Affiliations:** 1 Laboratory of Biopolymers and Pilot Plant of Bioprocessing of Agro-Industrial and Food By-Products, Biotechnology Department, Universidad Autónoma Metropolitana, Av. San Rafael Atlixco No. 186. Col. Vicentina, C.P., 09340 Mexico City, Mexico; shln1990@hotmail.com (L.N.S.); monclopz_qa@hotmail.com (M.L.); godzypotzy@hotmail.com (E.M.-D.); d_andrewriel@hotmail.com (A.E.); 2 Departamento de Alimentos y Biotecnología, Facultad de Química, Universidad Nacional Autónoma de México, 04510 Mexico City, Mexico; tecante@unam.mx (A.T.); mgimeno@unam.mx (M.G.)

**Keywords:** fresh cheese, *Listeria monocytogenes*, chitosan, grafting, lactic acid

## Abstract

A chitosan from biologically obtained chitin was successfully grafted with d,l-lactic acid (LA) in aqueous media using *p*-toluenesulfonic acid as catalyst to obtain a non-toxic, biodegradable packaging material that was characterized using scanning electron microscopy, water vapor permeability, and relative humidity (RH) losses. Additionally, the grafting in chitosan with LA produced films with improved mechanical properties. This material successfully extended the shelf life of fresh cheese and inhibited the growth of *Listeria monocytogenes* during 14 days at 4 °C and 22% RH, whereby inoculated samples with chitosan-*g*-LA packaging presented full bacterial inhibition. The results were compared to control samples and commercial low-density polyethylene packaging.

## 1. Introduction

*Listeria monocytogenes* is a foodborne pathogen that affects mainly the gastrointestinal tract and also potentially causes septicemia, meningitis, encephalitis, spontaneous abortion or stillbirth [1,2]. Listeriosis can be a severe disease, particularly among susceptible populations and a leading cause of death by foodborne illness [3,4,5]. Outbreaks have been strongly associated with cheese consumption. Fresh cheeses are usually protected from contamination by refrigeration and usually packed with paper or shrink-wrapped with polyethylene or polypropylene with potential risk of proliferation of aerobic mesophilic bacteria, coliforms, molds or yeast. Therefore, these cheeses are usually consumed within one month after manufacture with mounting losses of sensory characteristics throughout this period. The most sensitive of these types of products are soft paste unpressed fresh cheeses. They usually have short shelf lives of up to 10 days and rapid perishability owing to their high moisture (45%–55%) and low salt contents and neutral pH (6.0–6.5) [6]. Additionally, when made from raw milk fresh cheese contains a wide variety of microorganisms, which can include human pathogens as *L. monocytogenes*, which outbreaks in México have been associated with consumption of this type of cheeses [7].

Recent studies in food packaging are tackling these issues and antimicrobial materials with adequate properties have proved successful to reduce the growth of undesirable microorganisms and to preserve cheese qualities, allowing shelf life to be extended while maintaining good product quality and safety [8]. According to the current international regulations, these packaging materials must be innocuous, resistant and not react or alter the chemical, nutritional and sensorial properties of the packaged products. The food industry is focused on the development of non-toxic antimicrobial packaging options following the increasing consumer demand for biodegradable and in some cases even edible coatings free of preservatives or potentially toxic chemical additives that can migrate into foods. There has been increasing interest in antimicrobial films because microbial contamination occurs mainly on the cheese surface, which is the primary spoilage source. Films derived from renewable biomass sources and so-called bioplastics, such as chitosan-based films, might be potential alternatives to oil-derived plastics [9]. Chitosan is a non-toxic, renewable, biodegradable and biocompatible polysaccharide mainly composed of repeating glucosamine units. However, chitosan films present poor mechanical properties, which restrict their application for food packaging. As a way to avoid this drawback, grafting with other components can improve its mechanical properties [9]. This study establishes a method for extending the shelf life of fresh cheeses with inhibition of *L. monocytogenes* by using packaging films of chitosan grafted with d,l-lactic acid (LA).

## 2. Results and Discussion

### 2.1. Grafting of LA onto Chitosan

Table 1 shows the yields, degree of grafting and some properties of the samples. The ChLA-TSA film sample had a thickness of *ca.* 0.15 mm, which has a positive effect on the WVP (4.2 × 10^5^ g·mm/m^2^·h·kPa) at the studied RH interval (22%–75%). Noteworthy, Di Pierro *et al.* [10] reported WVPs up to 0.0167 g·mm/m^2^·h·kPa which are below the 0.2613 g·mm/m^2^·h·kPa reported by Miranda *et al.* [11]. However, despite the WVP values reported for chitosan films, a comparison between data is inaccurate because the permeability of the hydrophilic films is directly related to the interval of RH, film thickness and the method employed [11]. Another factor that influences the WVP is the addition of plasticizers that generally favors the migration of water molecules [12]. However, no significant differences were found on WVP with the inclusion of PSI in our ChLA-TSA films (Table 1).

**Figure 1:**
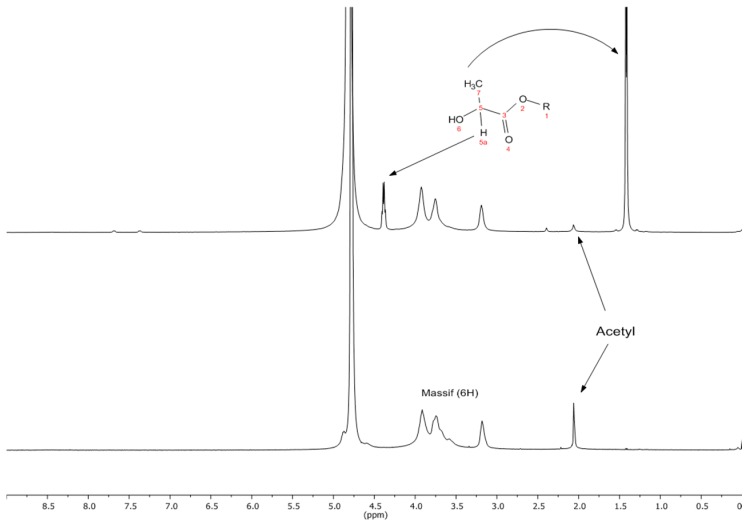
^1^H-NMR spectra of Ch (**bottom**) and ChLA-TSA (**top**).

The characterization of the obtained chitosan gave an *M*_v_ of 274.8 ± 5 kDa, ash content of 1 ± 0.05 and a DA of 6.43 ± 0.19 for this polymer. The FT-IR spectrum of the grafted chitosan shows the characteristic signals for chitosan and that at *ca.* 1700 cm^−1^ assigned to the carbonyl moiety of LA [13,14] (see the Supplementary Data file for the FTIR spectra of chitosan and grafted chitosan samples). The ^1^H-NMR spectra of the Ch and grafted sample ChLA-TSA (Table 1) film are shown in Figure 1 (bottom spectrum) and Figure 1 (top spectrum), respectively, with the assigned signals.

### 2.2. Mechanical Properties of Films

The mechanical properties in films are shown in Table 2, where is clearly demonstrated that the flexibility of the grafted materials was improved compared to that of Ch films. The decrease of the Young’s modulus indicates that the film is less rigid and possesses an enhanced capacity for elongation, which is an interesting feature for some applications. In general, the mechanical properties on chitosan-based films are affected by the chitin source, film thickness, DA and *M*_v_ of the chitosan, the use of plasticizers as well as the initial chitosan acid solution [15]. Several studies suggest that the films prepared with acetic acid are stiffer owing to the existence of relatively strong intermolecular interactions than those formed with citric acid [16]. In addition, grafting can also affect the mechanical properties.

### 2.3. Analyzes of Fresh Cheese Inoculated with L. monocytogenes

**Figure 2:**
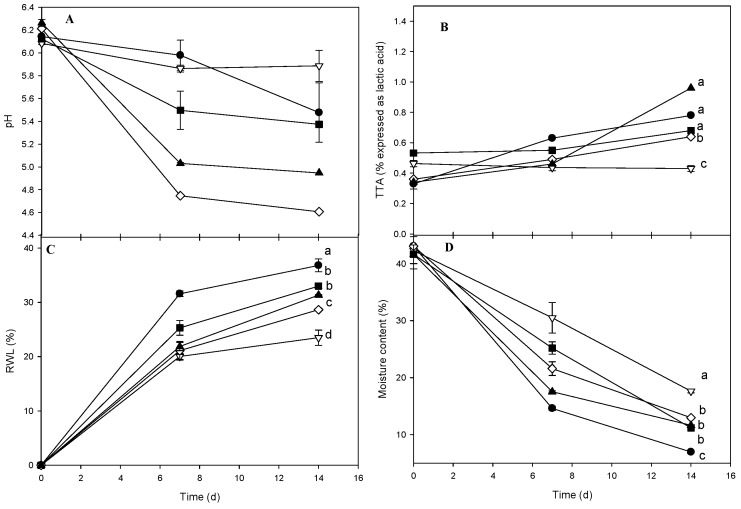
Time course of pH (**A**); TTA (**B**); RWL (**C**) and moisture content (**D**) of chesses inoculated with *L. monocytogenes* stored at 4 °C and 22% RH coated with different materials. Control (uncoated) (●); LDPE (▽); ChLA (■); ChLA-TSA (◊) and ChLA-TSA-SPI (▲). Data are the average of three determinations. Plots with the same letter in a graph are not significantly different at *p* < 0.05 as determined by multiple comparisons of means by the Tukey-Kramer test. Continuous lines are included only as a visual guide.

The initial moisture of samples was about 45% and its decrease was near 5% for the control sample after 14 days (Figure 2D). All samples displayed a considerable moisture loss but the LDPE packaging exhibited the lowest (20%) after 14 days in a statistically different group. This behavior is attributed to low moisture transfer and small VWP of the LDPE film. In addtion, after 14 days of storage, the control and grafted cheeses show significant differences in moisture content. Noteworthily, the inoculation procedure required that cheese samples remain for 1 h in a laminar flow hood under ambient aseptic conditions which might favor the draining of serum.

**Figure 3:**
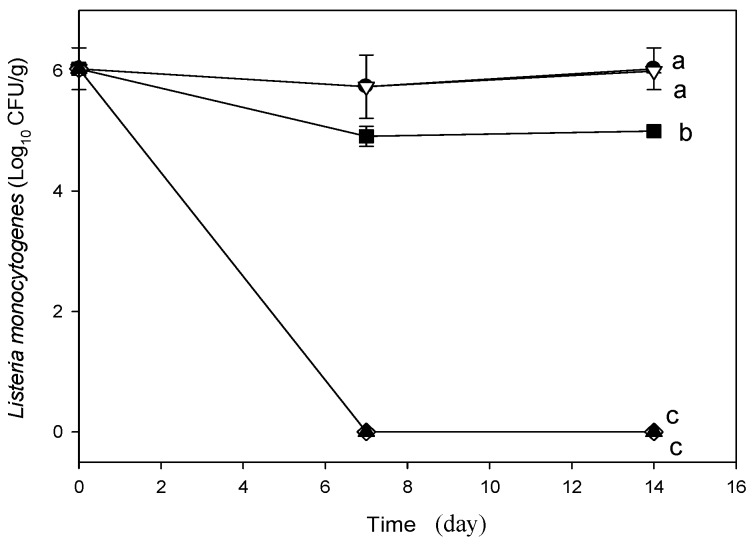
Time course of plate count with Oxford agar of *L. monocytogenes* in cheese at 4 °C and 22% RH. Symbols: Control (uncoated) (●); LDPE (▽); ChLA (■); ChLA-TSA (◊) and ChLA-TSA-SPI (▲) Data are the average of three determinations. Plots with the same letter in the graph are not significantly different at *p* < 0.05 as determined by multiple comparisons of means of microbial counts between materials by Tukey-Kramer test.

In regard to RWL (Figure 2C), significant differences among treatments were also observed. Cheese packed in LDPE had the lowest value (0.5% ± 0.8%) while the control sample displayed the poorest results. Samples wrapped with ChLA-TSA film showed the best results among the biodegradable packaging examined with statistically less RWL than that with ChLA. This experimental result might be attributed to the highest presence of grafted LA in the ChLA-TSA material. Nonetheless, the low RWL in the LDPE sample [17] compared with chitosan-based materials was expected owing to the WVP values as well as the relatively high RWL (40%) in the control after 14 days of storage. The pH of cheeses infected with *L. monocytogenes* tends to decrease during storage for all package types, although this was less noticeable for LDPE followed by the control samples (Figure 2A). The LDPE and control packages showed a significant difference from all other experimental results. The pH for LDPE was about 6 and remained nearly constant from day 7 to 14 owing to the reduced migration of acids throughout the film. Notably, ChLA-TSA packaging showed the lowest pH values after 14 days notwithstanding the observed TTA increase (Figure 2B). In this regard, Martins *et al.* [18] compared the pH of Ricotta cheese inoculated with *L. monocytogenes* and coated with galactomannans containing nisin to demonstrate that coated cheeses had lower pH values at the end of storage than that of control without coating. The authors attributed this behavior in the control due to the release of alkaline components generated during protein degradation by surface microorganisms. However, we did not observed a pH decrease under any condition, thereby proteolytic activities might be ruled out in our study. After 14 days of storage, the TTA of coated cheeses with ChLA-TSA-SPI exhibited a significant increase from ca. 0.35 to near 1 (Figure 2B). This increase might be ascribed to a favored LA migration. Statistical analysis however, shows no significant differences in the TTA for ChLA-TSA-SPI, control and ChLA but for ChLA-TSA. Therefore, the experimental results evidenced that the presence of the plasticizer did not affect LA migration significantly. Worth of note, LDPE packaging tend to decrease the TTA of cheese samples thorough the storage. On the other hand, the TTA increase measured in the control, which is remarkable from 7 to 14 days, might indicate the formation of LA due to the lactose fermentation by lactic acid bacteria (LAB). In regard to the microbiological analysis, there was no microbial count of *L. monocytogenes* in the non-inoculated cheeses. On the other hand, the concentration of *L. monocytogenes* in inoculated cheese was 1 × 10^4^ CFU/mL, however, no growth was observed during the first 7 days. Lactic bacteria might possess the ability to inhibit this microorganism due to the decrease in pH owing to the production of lactic acid or due to competition for utilization of carbohydrates in the substrate that leads to decrease the common specific nutrients of *L. monocytogenes*. In addition, lactic acid bacteria’s ability to produce inhibitory compounds such as hydrogen peroxide, bacteriocins and bacteriocin-like substances is well known [18]. Nonetheless, after 14 days of storage we observed characteristic *L. monocytogenes* colonies in LDPE coated and control samples (Figure 3). Our results suggest that the coated cheeses with chitosan-based packaging have the lowest bacterial count significant differences with respect to the control and LDPE samples. Interestingly, the samples coated with ChLA-TSA and ChLA-TSA-SPI showed complete inhibition of *L. monocytogenes* after 7 days of storage and this was statistically different from the result of ChLA. The loss of moisture and the decreased water activity (a_w_) could limit the growth of *L. monocytogenes,* for which the minimum a_w_ for growth is 0.92 [19]. Nonetheless, despite the significant differences in the measured moisture (Figure 2D) between the uncoated control and coated cheeses (Figure 2), there is a conclusive evidence of the pathogen depletion by the grafted chitosan films (Figure 3). In the aforementioned study by Martins *et al.* [18], the growth of *Listeria monocytogenes* in Ricotta cheese using galactomannan with added nisin was 4.5 log CFU/g after 15 days of storage. This value means less inhibition of this pathogen than the measured in our fresh cheese using grafted chitosan packaging (Figure 3).

### 2.4. SEM Analyses of Samples

**Figure 4:**
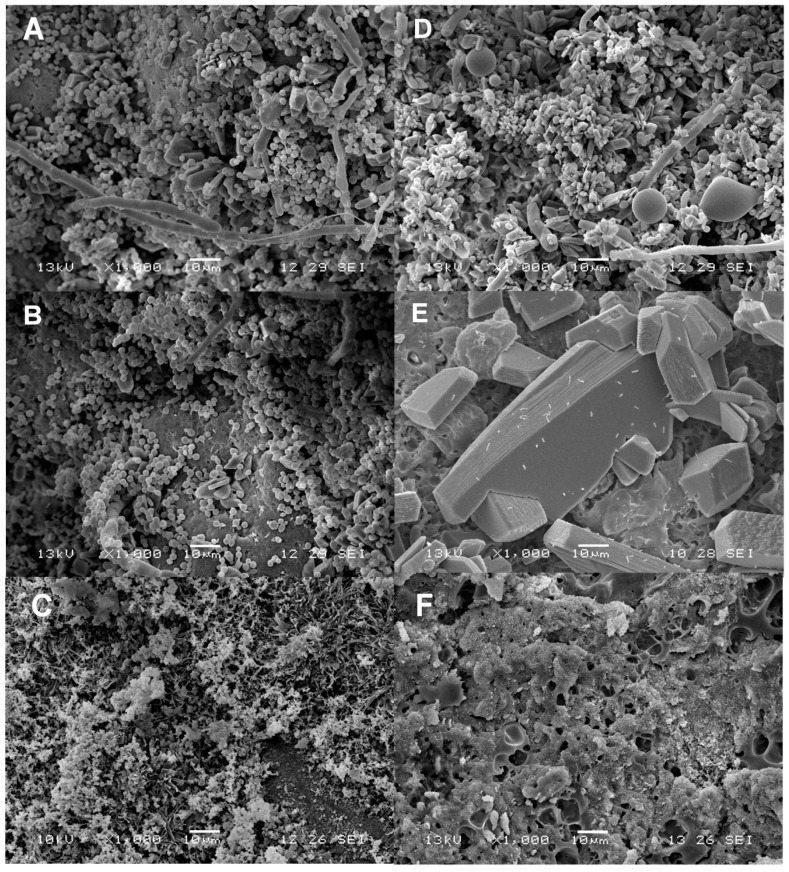
SEM micrographs of fresh cheese samples non-inoculated:control non-packaged (**A**); LDPE packaging (**B**); ChLA-TSA-SPI packaging(**C**); Inoculated with *L. monocytogenes* control non-packaged (**D**); LDPE packaging (**E**) and ChLA-TSA-SPI packaging (**F**).

Figure 4 shows SEM micrographs of coated cheese samples that were not inoculated with *L. monocytogenes* after 14 days of storage. Figure 4A corresponds to the uncoated control, in which a segment of hyphae and spores are observed due to fungal growth, as well as yeast proliferation. These observations are well related with the high count (9.3 log CFU/g) of fungi and yeast seen in this study in potato dextrose agar. Several fungi and yeasts of the genera *Penicillium* sp., *Cladosporium* sp. and *Geotrichum candidum* have been identified in Mexican fresh cheeses and in the case of yeast the presence of *Candida guilliermondii*, *Candida lipolytica* and *Candida tropicalis* has been detected [20].

The non-inoculated cheeses packed in LDPE are shown in Figure 4B where it is seen that this material displayed low yeast inhibition effectiveness. The growth of filamentous fungi is evident in addition to rod-shaped bacteria. Contrarily, SEM micrographs of samples of cheeses packaged with grafted ChLA-TSA-SPI, shown in Figure 4C, display a considerable reduction of microorganisms and hyphae and spores and yeast are not observed. The spongy complex formed by agglomeration of spherical particles might correspond to casein micelles. They are reported to display spongy and porous structures with diameters between 30 and 300 nm [21]. Figure 4D–F correspond to the SEM of inoculated cheese samples with *L. monocytogenes* (see the Supplementary Data for SEM images of *L. monocytogenes*). The SEM for inoculated cheese control samples show a segment of hyphae and fat globules (Figure 4D). Fat globules of milk have a diameter ranging up to 2–6 μm thus being 25 times larger than the average diameter of casein micelles [21], which is consistent with our SEM images of fresh cheeses. The presence of short rods and bacilli, both less than 5 μm in size, are observed with shaped sheet structures in Figure 4E for LDPE coated samples. These crystals might be attributed to lactose, in agreement with SEM studies conducted by Kougoulos *et al.* using anti-solvent crystallization techniques [22]. In addition, spongy and porous structures corresponding to the casein micelles are present.

Remarkably, bacteria are also observed as short rods with morphology similar to *L. monocytogenes* in the inoculated cheeses in Figure 4D,E. On the other hand, Figure 4F shows inoculated cheeses coated with ChLA-TSA-SPI, which was found free of visible microorganisms. Noteworthily, the presence of lactose crystals and also shrunk casein micelles is also observed in cheese samples coated with the chitosan-based material without bacterial inoculation (Figure 4C). In this regard, Martin *et al.* [21] reported casein micelle distortion as a consequence of the drying step during SEM sample preparation. In some samples (Figure 4F) differences in morphology are observed, which might be due to interactions between aggregates of casein and chitosan. In this regard, it has been documented that casein and chitosan can interact to form soluble or insoluble complexes following Maillard reaction or ionic interactions, which has found several applications including drug delivery [23].

## 3. Materials and Methods

### 3.1. Materials

Chitosan was obtained by heterogeneous deacetylation of chitin extracted from the lactic acid fermentation (LAF) of shrimp (*Litopenaeus vanameii*) wastes and purified as reported elsewhere [10]. LA (85% aqueous solution) was acquired from J.T. Baker (Toluca, Mexico). Triethanolamine (TEA) and *p*-toluenesulfonic acid (TSA) was supplied by Sigma-Aldrich (Saint Louis, MO, USA). Low-density polyethylene (LDPE) film samples were kindly donated by Casa Miyako (Mexico City, Mexico). The *L. monocytogenes* strain was a kind gift of Dr. Rigoberto Hernández-Castro from the culture collection of Hospital General Dr. Manuel Gea González (Mexico City, Mexico) and in trypticaseine soy broth (TSB) at 37 °C for 24 h to attain 10^7^ colony-forming units (CFU) per mL. Inoculum was prepared by dilution in sterile saline solution (0.9 NaCl *wt*/*v*% (weight/volume)) up to 10^4^ CFU/mL. Soy protein isolate (SPI) was purchased to Sigma Aldrich. Fresh cheeses were provided by a farm (Celaya Guanajuato, Mexico).

### 3.2. Preparation of Chitosan Films

Chitosan was dissolved (1.15 *wt*/*v*%) in acetic acid (0.1 M) under continuous agitation for 24 h at ambient temperature. Solutions were filtered through a metallic sieve (mesh No. 100) to remove insoluble matter. Individual chitosan films (Ch) were obtained by casting as follows: 30 g of the chitosan solution were poured into polystyrene (PS) Petri dishes and dried in an oven at 60 °C for 24 h.

### 3.3. Grafting of Chitosan with LA and Production Films

The grafting of chitosan with LA was conducted following the method reported by Albertsson and co-workers [13] with some modifications. In a typical experiment, 40 mL of a chitosan (1 g) solution in LA (0.5 M) was mixed with 160 mL of LA (0.3 M) containing TSA (1% *w*/*w*) and heated at 80 °C for 2 h under inert atmosphere. Then, solutions were poured into PS Petri dishes, placed in an oven (80 °C) under vacuum for 3 h and further dried at atmospheric pressure for 6 h at 60 °C to give film samples (ChLA-TSA). The non-reacted LA in the resulting films was removed by Soxhlet extraction using acetone for 18 h. In another formulation, SPI aqueous solution (0.25 *wt*/*v*%) was added at 25 °C to the grafted chitosan after the casting procedure. Then, the ChLA-TSA-SPI films were obtained by casting 50 g of the solution onto PS Petri dishes placed in an oven at 60 °C for 24 h [24]. Blank experiments were conducted using identical procedure without the TSA addition to obtain the ChLA samples.

### 3.4. Chitosan Characterization and Characterization of Films

The *M*_v_ of chitosan, dissolved in acetic acid (0.3 M) and sodium acetate (0.2 M) at 25 °C [25], was determined from time flow measurements in an Oswald glass capillary viscometer using the Mark-Houwink-Kuhn-Sakurada equation with α = 0.85 and *K* = 1.38 × 10^−5^ L/g. Degree of acetylation (DA) was determined by integration of characteristic signals in the ^1^H-NMR spectra recorded in D_2_O with 10% HCl in a spectrometer (AVANCE-III 500, Bruker, Rheinstetten, Germany) [14,25]. The degree of grafting percentage (DG%) was calculated according to Equation (1):

(1) $$\text{DG}\left(\%\right)=\left(\frac{100\left(W_{2}-W_{1}\right)}{W_{1}}\right)$$

where W_1_ and W_2_ are the weights of the materials on a dry basis before and after the grafting procedure, respectively.

Infrared spectra were acquired in an ATR-FTIR apparatus (Perkin Elmer 100, Waltham, MA, USA). Mechanical properties upon extension were determined according to ASTM D882-97 [26] in a mechanical testing machine (SINTECH 1/S, MTS, Eden Prairie, MN, USA) with a load cell of 100 N. Samples were conditioned to 44% relative humidity (RH) at 25 °C prior to analyses. Water vapor permeability (WVP) was determined at 4 °C using a modification of the ASTM method [27]. Films were cut to a diameter of 2 cm and then fixed to permeability cells. Storage conditions of fresh cheese were simulated within an interval of 22%–75% RH. The cell with an RH of 75% was prepared with a saturated solution of NaCl and the RH in the chamber was 22% by a saturated solution of KCH_3_CO_2_. Determinations were conducted by triplicate. Determination of film thickness was conducted by six random measurements to each sample with a digital micrometer (Mitutoyo 1D-C112E, Kanagawa, Japan).

### 3.5. Analysis of Coated Cheese Samples

*L. monocytogenes* inoculum (35 mL) with 10^4^ CFU/mL was poured onto a sterile wooden board of 1050 cm^2^ surface previously sterilized in an autoclave [3]. Cheese samples were placed on the inoculated board for 30 min. Then, the inoculation procedure was repeated with an additional 35 mL of *L. monocytogenes* suspension and the cheese samples were put on their non-inoculated sides for 30 min. The inoculated cheese samples were coated with the chitosan-based materials and LDPE. Films were heat sealed using gelatin as a sealing lacquer. The inoculation procedure and coating of samples were carried out under aseptic conditions using a biological safety cabinet. Control and cheeses coated with films were stored at 22% RH and 4 °C.

For weight loss determinations, samples of cheese coated with films and that for control were weighed daily. The slope of the weight loss *vs.* time plot was divided by the exposed film area to calculate the water vapor transmission rate. Relative weight loss (RWL) in cheese samples was determined by measuring the weight of samples at the beginning (*W*_I_) and after 7 and 15 days of storage (*W*_F_) using Equation (2):

(2) $$RWL=\frac{\left(W_{I}-W_{F}\right)}{W_{I}}\times 100$$

For pH and acidity determinations, cheese samples were unpacked every 7 days, then 10 g were suspended in 100 mL of distilled water and mixed during 2 min in a homogenizer (Sper Scientific 460003, St. Louis, MO, USA). Suspensions were filtered, and supernatants analyzed to determine total acidity (TTA) by potentiometric titration using 0.1 N NaOH. Results were expressed as lactic acid percentage. The pH measurements were carried out in a digital potentiometer (Hanna Instruments Digital, Padova, Italy). Moisture content was determined in a thermobalance (Wiggen Hauser, Berlin, Germany) at 80 °C using 1 g of material. *L. monocytogenes* enumeration was carried out by inoculation of decimal dilutions in peptone water on Oxford agar base plates and incubation at 30 °C for 48 h. The black colonies were counted and expressed as Log_10_ CFU/g. For yeasts and molds enumeration, serial dilutions were prepared in sterile saline solution and inoculated on plates of potato dextrose agar acidified with tartaric acid. Determinations were done by triplicate.

### 3.6. Scanning Electron Microscopy Analyses

Square (0.5 cm × 0.5 cm) samples from the surface of the cheeses were fixed with 5% (*v*/*v*) glutaraldehyde and stored for 24 h at 4 °C. Samples were washed with phosphate buffer (0.1 M pH 7) and subsequently treated with 1% (*w*/*v*) OsO_4_ for 2 h. Then, samples were dehydrated with alcohol and sputtered with carbon and gold for examination in a scanning electron microscope (JEOL JSM-5900 LV, Tokyo, Japan).

### 3.7. Statistical Analysis

A randomized design was carried out in triplicate for experiments with chitosan-based packaging materials and LDPE. NCSS 2000 software (NCSS Inc., Kaysville, UT, USA) computed the analysis of variance with yield, degree of grafting, film thickness, WVP, mechanical properties, RWL, moisture content, pH, TTA and *L. monocytogenes* counts as response variables. Means were compared with Tukey-Kramer multiple means comparison test (*p* < 0.05).

## 4. Conclusions

This work demonstrates that the use of non-toxic chitosan-*g*-LA for packaging of fresh cheese offers an alternative for the preservation of the quality of this product during storage and increased shelf life and more importantly it inhibits the growth of *Listeria monocytogenes*, which is considered a very dangerous human food pathogen. Our chitosan-based material displayed a remarkable improvement compared to non-coated cheeses as well as LDPE coated samples, which represents an ecological advance for reduction of non-degradable oil-derived material use. Additionally, the chitosan grafted with LA displays improved mechanical properties as compared to native chitosan.

## Figures and Tables

**Table 1:** Experimental data for chitosan and chitosan-grafted LA.

Material	Yield (%)	Degree of Grafting (%)	Film Thickness (mm)	VWP (g·mm/m^2^·h·kPa)
Ch	-	-	0.062 ± 0.006 ^b^	7 × 10^−^2 ± 2 × 10^−3 a^
ChLA	51.16 ± 1.96 ^a^	36.85 ± 2.33 ^b^	0.074 ± 0.003 ^b^	1.6 × 10^−3^ ± 2.6 × 10^−4 b^
ChLA-TSA	58.77 ± 1.65 ^a^	43.62 ± 0.90 ^a^	0.15 ± 0.003 ^a^	4.2 × 10^−5^ ± 1.4 × 10^−6 c^
ChLA-TSA-SPI	48.45 ± 0.58 ^a^	33.38 ± 0.80 ^b^	0.15 ± 0.021 ^a^	4.4 × 10^−5^ ± 3.1 × 10^−6 c^
LDPE	-	-	2.07 ± 0.3 ^c^	7.6 × 10^−10^ ± 1.4 × 10^−9 d^

Data are shown as the average of four determinations with their corresponding standard deviation. Values in a column with the same letter are not significantly different at *p* < 0.05 determined by multiple comparisons of means by Tukey-Kramer test.

**Table 2:** Mechanical properties of the prepared films.

Material	Young’s Modulus (MPa)	Elongation at Break (%)	Tensile Strength (MPa)
Ch	2.2 ± 0.56 ^d^	57.2 ± 27.0 ^b^	0.61 ± 0.20 ^c^
ChLA	16.8 ± 0.20 ^c^	75.4 ± 5.9 ^b^	31.3 ± 1.12 ^b^
ChLA-TSA	105.6 ± 2.15 ^b^	67.6 ± 5.6 ^b^	19.25 ± 1.19 ^b^
ChLA-TSA-SPI	49.26 ± 7.43 ^c^	78.0 ± 9.5 ^b^	88.29 ± 2.46 ^a^
LDPE	371.62 ± 12.48 ^a^	205 ± 24.4 ^a^	23.58 ± 0.96 ^b^

Data are shown as the average of four determinations with their corresponding standard deviation. Values in a column with the same letter are not significantly different at *p* < 0.05 determined by multiple comparisons of means by Tukey-Kramer test.

## Supplementary Materials

Supplementary materials can be accessed at: http://www.mdpi.com/1420-3049/21/4/469/s1.
