# Supplementary material for: Inhibition of Listeria monocytogenes in Fresh Cheese Using Chitosan-Grafted Lactic Acid Packaging
Source: Molecules. 2016 Apr 8;21(4):469. doi: 10.3390/molecules21040469 (PMC6273688; doi:10.3390/molecules21040469)
Supplement: Supplementary file 1 [file molecules-21-00469-s001.pdf]

Supplementary Materials: Inhibition of *Listeria monocytogenes* in Fresh Cheese Using Chitosan-Grafted Lactic Acid Packaging

Laura N. Sandoval, Monserrat López, Elizabeth Montes-Díaz, Andres Espadín, Alberto Tecante, Miquel Gimeno and Keiko Shirai

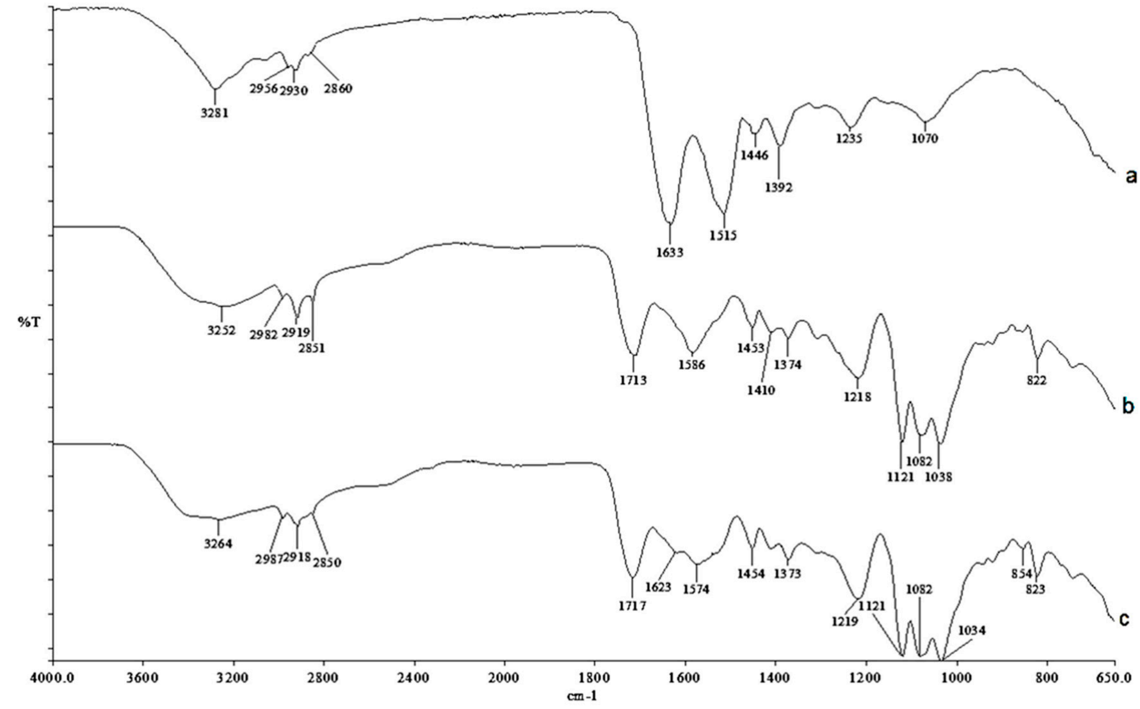

Figure S1. FTIR spectra of samples (**a**) SPI; (**b**) ChLA; (**c**) ChLA-TSA-SPI.

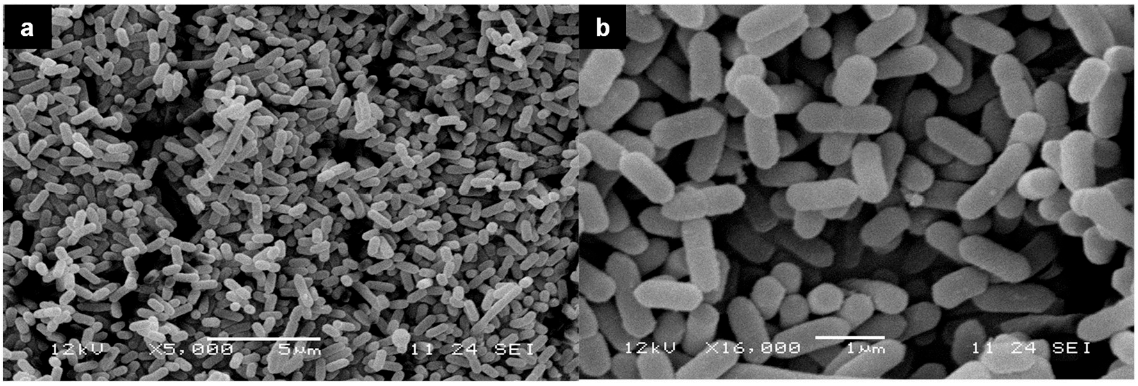

Figure S2. SEM of *Listeria monocytogenes* inoculum cultured in TSB at 37 °C for 24 h and diluted in sterile saline solution.
